# ROCK signaling at the interface of fibrosis and neurodegeneration: mechanisms and therapeutic potential

**DOI:** 10.3389/fcell.2026.1933409

**Published:** 2026-10-30

**Authors:** Jeysson Sanchez-Suarez, Yvonne Adu-Rutledge, Kunho Bae, Hyungwoo Lee, Anton Lennikov, Nakyung Ryoo, Anil Upreti, Aruvi Vijikumar, William P. Miller, Leo A. Kim

**Affiliations:** 1 Schepens Eye Research Institute of Massachusetts Eye and Ear Infirmary, Boston, MA, United States; 2 Department of Ophthalmology, Harvard Medical School, Boston, MA, United States; 3 Department of Ophthalmology, Biomedical Research Institute, Seoul National University Hospital, Seoul, Republic of Korea; 4 Department of Ophthalmology, Konkuk University School of Medicine, Konkuk University Medical Center, Seoul, Republic of Korea; 5 Department of Ophthalmology and Visual Neuroscience, University of Minnesota, Minneapolis, MN, United States

**Keywords:** fibrosis, mechanotransduction, MRTF/SRF, neurodegeneration, rho-associated kinase, ROCK1, ROCK2, YAP/TAZ

## Abstract

Rho-associated coiled-coil containing protein kinases 1 and 2 (ROCK1 and ROCK2) regulate cytoskeletal dynamics, actomyosin contractility, extracellular matrix remodeling, and responses to mechanical stress. In the eye and central nervous system (CNS), these functions intersect with fibrocontractile scarring, neuronal dysfunction, impaired regeneration, and neuroinflammation. This review examines ROCK signaling at the interface of ocular fibrosis and neurodegeneration, emphasizing the retina as a setting in which neural injury and pathological remodeling can coexist. We outline the physiological and pathological consequences of ROCK-mediated mechanotransduction, using peripheral fibrosis only as brief comparative context, and examine its relationship with MRTF/SRF and YAP/TAZ transcriptional modules. We then assess evidence from ocular fibrotic disease, axonal regeneration, synaptic plasticity, microglial activation, and CNS injury-associated scarring, distinguishing direct ROCK-dependent mechanisms from extrapolation across tissues. ROCK1 and ROCK2 display overlapping functions with context-dependent specialization, but current evidence does not support a universal division of labor between the isoforms. Single-cell, spatial, and multi-omics approaches offer opportunities to identify disease-relevant cell populations, although transcript abundance alone cannot establish kinase activity or therapeutic dependence. Finally, we consider ocular and neural therapeutic development, including isoform-informed targeting, local delivery, and mechanism-aligned combinations. The central challenge is to suppress persistent pathological remodeling while preserving physiological repair and beneficial neural or glial responses. Resolving the cellular, spatial, and temporal requirements for ROCK signaling will be essential for testing whether antifibrotic and neuroprotective effects can be achieved together.

## 1 Introduction

Fibrosis and neurodegeneration are traditionally regarded as distinct pathological processes. Fibrosis is characterized by excessive extracellular matrix (ECM) deposition, progressive tissue stiffening, disruption of tissue architecture, and ultimately organ dysfunction (Santos and Lagares, 2018), whereas neurodegenerative disorders are defined primarily by neuronal dysfunction, synaptic failure, impaired regenerative capacity, and progressive neuronal loss (Lepeta et al., 2016; Koch et al., 2018; Dejanovic et al., 2024; Gadhave et al., 2024; Aranda-Abreu et al., 2026). Despite these differences, fibrotic and neurodegenerative diseases share features of maladaptive cellular reprogramming, chronic stress, impaired homeostatic resilience, and progressive disruption of tissue organization (Koch et al., 2018; Santos and Lagares, 2018; Wang et al., 2018a; D'Ambrosi and Apolloni, 2020; Wareham et al., 2022). These parallels are particularly relevant in ocular and neural tissues, where structural, inflammatory, and biomechanical disturbances can interact to shape disease progression (Fernandez-Klett and Priller, 2014; Pogoda and Janmey, 2018; Santos and Lagares, 2018; D'Ambrosi and Apolloni, 2020; Pinter and Alpar, 2022; Lozinski et al., 2024).

The mechanical microenvironment provides an important framework for understanding this convergence. Cells continuously encounter physical cues such as ECM stiffness, tensile stretch, shear stress, and injury-associated changes in tissue architecture, which can influence cellular behavior through mechanotransduction (Santos and Lagares, 2018; Safa et al., 2022). These cues are especially relevant in the eye and central nervous system (CNS), where tissue organization is tightly linked to function. In the eye, altered biomechanics affect structures and cell populations including the trabecular meshwork, Schlemm’s canal, optic nerve head, Müller glia, and retinal vascular and fibrotic compartments (Safa et al., 2022). In the brain, changes in ECM organization and tissue mechanics have been associated with glial dysfunction, impaired regeneration, and neurodegenerative progression (Koch et al., 2018; Pogoda and Janmey, 2018). Although many mechanistic principles of force-responsive signaling have been established outside neural and ocular systems, they provide a useful framework for investigating how altered mechanics may contribute to disease in these tissues (Dupont et al., 2011; Loirand, 2015).

Rho-associated coiled-coil containing protein kinases 1 and 2 (ROCK1 and ROCK2), major downstream effectors of RhoA, are well positioned within this framework because they couple cytoskeletal remodeling to cellular tension and mechanosensitive transcription. ROCK regulates F-actin organization, myosin activity, focal adhesion dynamics, polarity, and motility, thereby influencing how cells generate and respond to mechanical forces (Amano et al., 2010a; Loirand, 2015). Through its effects on actin polymerization and actomyosin contractility, ROCK also interfaces with force-sensitive transcriptional regulators, particularly myocardin-related transcription factor/serum response factor (MRTF/SRF) and Yes-associated protein/transcriptional coactivator with PDZ-binding motif (YAP/TAZ), which can stabilize changes in cell state, matrix production, migration, and stress responses (Amano et al., 2010a; Dupont et al., 2011; Small, 2012; Foster et al., 2017). ROCK signaling can therefore be considered not only an effector of cytoskeletal contraction but also an important component of the signaling network that converts biomechanical cues into longer-lasting cellular programs.

Peripheral fibrosis establishes that ROCK-dependent contractility can reinforce ECM remodeling and inflammatory crosstalk (Loirand, 2015; Knipe et al., 2018; Santos and Lagares, 2018). These findings provide comparative mechanistic context, but do not establish equivalent disease mechanisms in ocular or neural tissues. In the CNS, ROCK has instead been associated with impaired axonal regeneration, synaptic dysfunction, glial activation, and neuroinflammation (Koch et al., 2018; Pogoda and Janmey, 2018). The relevant question is therefore which cytoskeletal and remodeling mechanisms are shared, and which depend on the cellular and anatomical setting.

In this review, we focus on ROCK signaling in ocular fibrosis and neurodegeneration, with CNS injury-associated scarring providing an additional context for examining interactions between matrix remodeling and neural repair. We first summarize ROCK-mediated mechanotransduction and its physiological limits, then examine ocular fibrocontractile pathology, neural dysfunction, and glial responses. We subsequently evaluate isoform specialization, cell-state-resolved approaches, and therapeutic strategies for ocular and neural disease. Throughout, we distinguish direct evidence in these tissues from mechanisms inferred from peripheral models.

## 2 ROCK mechanotransduction in homeostasis and pathological remodeling

### 2.1 Physiological repair and the transition to persistent remodeling

ROCK-mediated mechanotransduction is not intrinsically pathological. Under physiological conditions, spatially and temporally controlled RhoA-ROCK activity allows cells to sense changes in matrix rigidity, cell geometry, adhesion, and externally applied force and to translate these cues into cytoskeletal and transcriptional responses (McBeath et al., 2004; Pertz et al., 2006; Dupont et al., 2011; Guilluy et al., 2011). Through direct phosphorylation of myosin regulatory light chain and inhibitory phosphorylation of myosin phosphatase, ROCK increases actomyosin contractility, whereas ROCK–LIMK signaling inhibits cofilin-mediated actin depolymerization, thereby promoting actin-filament stabilization (Amano et al., 1996; Kimura et al., 1996; Maekawa et al., 1999). These processes contribute to cortical tension, cell shape, cell-cell and cell-matrix adhesion, epithelial and endothelial barrier integrity, smooth-muscle tone, and the maintenance of tissue architecture under mechanical load.

Following injury, controlled ROCK-dependent actin remodeling supports migration, epithelial closure, wound contraction, and provisional matrix organization (Vaezi et al., 2002; Tomasek et al., 2006; Gates et al., 2007; Yoneda et al., 2007). Corneal and cutaneous injury studies illustrate the contribution of RhoA-ROCK and mechanosensitive YAP/TAZ signaling to epithelial repair (Yin and Yu, 2008; Zhang et al., 2011; Lee et al., 2014b). As tissue integrity is restored, declining inflammatory and profibrotic signals, reduced mechanical tension, and deactivation or clearance of reparative myofibroblasts normally diminish ROCK-dependent mechanosignaling, helping limit a temporary repair response from progressing to persistent fibrosis.

When these resolution mechanisms fail, persistent inflammation, TGF-β signaling, excessive cell-generated tension, and matrix stiffening can extend contractile responses beyond the reparative phase (Huang et al., 2012; Frangogiannis, 2020). Matrix-dependent reinforcement of cytoskeletal and transcriptional signaling can then maintain activated, matrix-producing states (Liu et al., 2015; He et al., 2022). Therapeutic interpretation therefore requires attention to duration, localization, cell identity, and the stage of injury, rather than assuming that all ROCK activity is harmful.

### 2.2 Comparative principles from peripheral fibrosis

Studies of lung, liver, heart, and skin fibrosis establish several transferable principles: ROCK-dependent actomyosin tension supports myofibroblast activation, matrix production, and interactions between fibrogenic and inflammatory cells (Knipe et al., 2015). Human scleroderma fibroblasts provide evidence linking ROCK to myofibroblast differentiation and ECM production (Akhmetshina et al., 2008), while experimental pulmonary fibrosis implicates both ROCK1 and ROCK2 (Knipe et al., 2018; Wu et al., 2020). These findings support a framework in which contractility and matrix remodeling can reinforce persistent cellular activation, with isoform dependence varying by tissue. Their relevance to this review is comparative: they motivate investigation of similar processes in ocular fibrocontractile disease and CNS injury-associated remodeling, but do not prove that neurodegeneration is a fibrotic process or that the same downstream programs operate in neurons and glia. Direct evidence from the eye and CNS must determine where this framework applies.

### 2.3 Mechanical regulation in ocular and neural tissues

In the eye and CNS, disease-associated changes in ECM organization, tissue stiffness, and mechanical load can actively influence cell behavior rather than simply reflect established pathology. Pressure, stretch, shear, and matrix-derived constraints shape remodeling and stress responses across neural tissues (Pillai and Franze, 2024; Prieto-Lopez et al., 2024; Ethier and Herberg, 2025; Konig et al., 2025). In ocular disease, these cues affect the trabecular meshwork, Schlemm’s canal, Muller glia, and optic nerve head glia (Safa et al., 2022; Prieto-Lopez et al., 2024; Prieto-Lopez et al., 2025); in the brain, altered tissue mechanics have been linked to glial dysfunction, impaired regeneration, metabolic disturbance, and neurodegenerative progression (Pogoda and Janmey, 2018; Wang et al., 2018b; Bonvento and Bolanos, 2021; Pillai and Franze, 2024). These observations support the view that ocular and CNS disorders share mechanobiological principles, while retaining important tissue-specific differences (Rocha et al., 2022; Rusciano et al., 2025).

ROCK couples mechanical inputs to actomyosin contractility, focal adhesion dynamics, and cytoskeletal tension (Rocha et al., 2022; Rusciano et al., 2025). However, much of the causal evidence linking these events to persistent force-responsive transcription comes from non-neural systems. The ocular and CNS studies discussed below should therefore be evaluated according to whether they demonstrate ROCK dependence directly, identify downstream mechanosensitive responses, or provide only a plausible connection between the two.

### 2.4 YAP/TAZ and MRTF/SRF as transcriptional effectors of ROCK-mediated mechanotransduction

YAP and TAZ are force-responsive transcriptional regulators. Matrix stiffness, cell spreading, and cytoskeletal tension can favor their nuclear localization (Dupont et al., 2011), while force transmission to the nucleus can facilitate YAP entry through nuclear pores (Elosegui-Artola et al., 2017). MRTF/SRF provides a complementary route coupled to actin polymerization: binding to G-actin retains MRTF proteins in the cytoplasm, whereas reductions in the available G-actin pool permit nuclear translocation and SRF-dependent transcription (Olson and Nordheim, 2010). RhoA/ROCK-associated actin remodeling can thus connect mechanical responses to contractile and matrix-producing programs (Ni et al., 2013; Yokota et al., 2017; Yuan et al., 2018; Blomme et al., 2019; Xie et al., 2022; Knipe et al., 2023). These pathways are interconnected rather than uniquely controlled by ROCK; GPCR-RhoA signaling can engage both MRTF-A and YAP (Yu et al., 2016; Yu et al., 2020).

In ocular systems, support for this framework comes from studies of scleral remodeling and pterygium-associated fibrosis. Mechanical strain in scleral fibroblasts and TGF-β1 stimulation in human pterygium fibroblasts engage RhoA/ROCK-dependent MRTF-A/SRF signaling that promotes myofibroblast differentiation and profibrotic responses (Yuan et al., 2018; Xie et al., 2022). Direct ocular evidence connecting ROCK-dependent mechanics to YAP/TAZ is less extensive in the literature assembled here, although broader mechanobiology studies support YAP/TAZ as a plausible mediator of force-sensitive remodeling (Kang et al., 2020; Li et al., 2020; Purdy et al., 2020). This evidence imbalance is important: the general ROCK-YAP/TAZ-MRTF/SRF architecture is well supported across experimental systems, but its cell-state-specific organization remains incompletely mapped in the eye and CNS.

The therapeutic implications depend on the responding cell population. Inhibiting a persistent contractile program in ocular fibroblasts may be useful, whereas suppressing transcriptional responses that support neuronal or glial repair may be detrimental. The distinction between direct ROCK dependence and broader YAP/TAZ or MRTF/SRF activity is therefore important when interpreting the ocular/CNS framework summarized in Figure 1.

**FIGURE 1:**
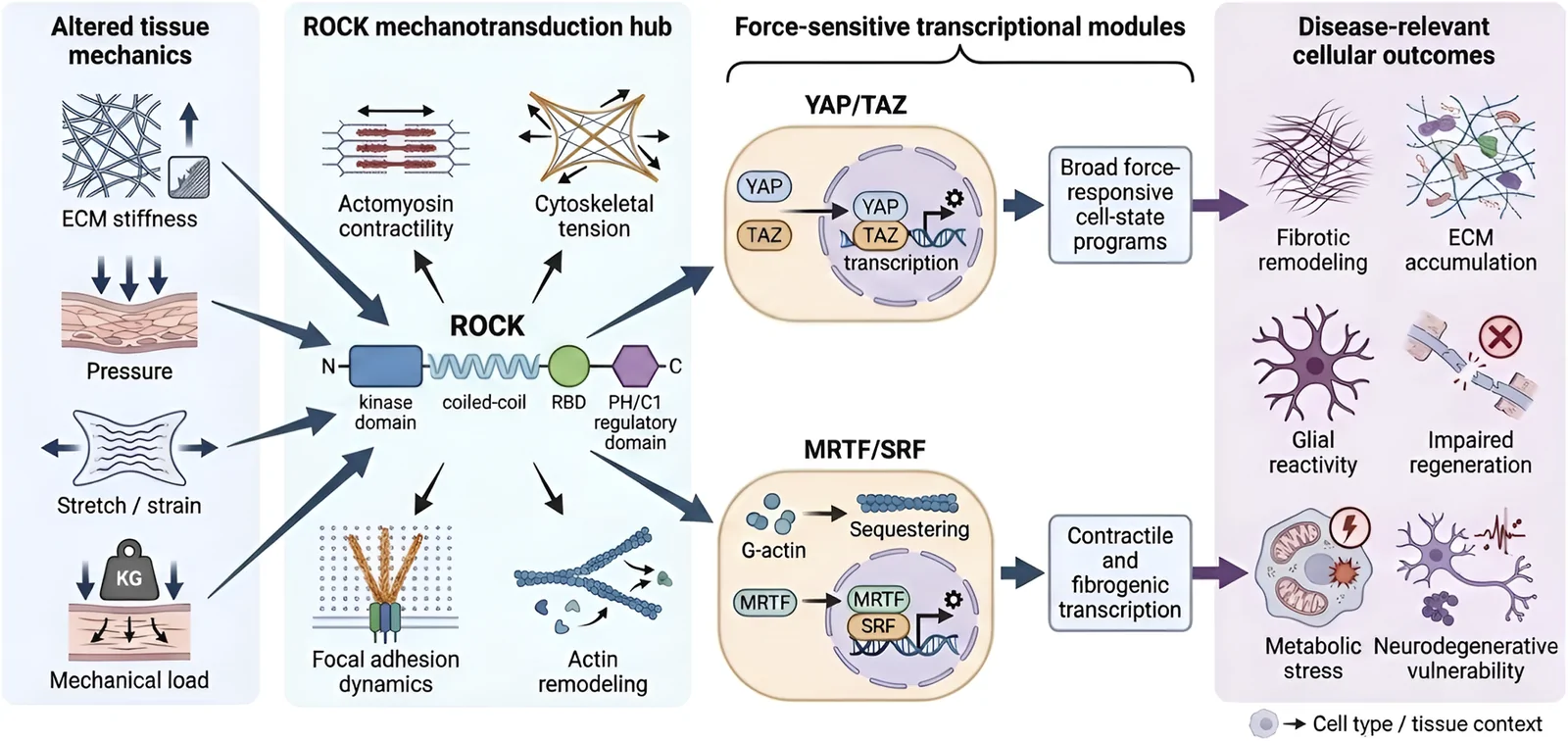
ROCK-dependent mechanotransduction links fibrosis and neurodegeneration across ocular and central nervous system tissues. This illustrates how disease-associated mechanical cues, including ECM stiffness, pressure, stretch/strain, and mechanical load, converge on ROCK signaling as a central mechanotransduction hub. ROCK integrates these inputs through regulation of actomyosin contractility, focal adhesion dynamics, actin remodeling, and cytoskeletal tension. These mechanical signals are transmitted to force-sensitive transcriptional modules, including YAP/TAZ and MRTF/SRF. YAP/TAZ activation supports broad force-responsive cell-state programs, whereas MRTF/SRF links actin remodeling to contractile and fibrogenic transcription. Depending on cell type and tissue context, these ROCK-dependent programs can promote disease-relevant outcomes such as fibrotic remodeling, ECM accumulation, glial reactivity, impaired regeneration, metabolic stress, and neurodegenerative vulnerability. Together, the figure highlights ROCK signaling as a shared mechanobiological axis connecting fibrosis and neurodegeneration across ocular and central nervous system tissues.

Therapeutically, YAP/TAZ and MRTF/SRF should not be viewed as independent terminal branches of ROCK signaling. YAP/TAZ-directed approaches may be most relevant when broad force-responsive transcription predominates, whereas MRTF/SRF-directed approaches may be favored when actin-dependent fibrogenic programming is dominant. Combined modulation may be appropriate when both pathways are concurrently engaged. However, comparative evidence establishing when single-versus dual-pathway targeting is superior remains limited. In neural and reparative glial populations, YAP/TAZ or MRTF/SRF activity may instead support regeneration or tissue repair, indicating that cell identity and disease stage may determine whether these pathways should be inhibited, preserved, or activated.

## 3 ROCK signaling in ocular fibrosis and retinal injury

### 3.1 Fibrocontractile remodeling and proliferative vitreoretinopathy

The convergence of neural and remodeling functions is particularly relevant in the retina, where neuronal injury and fibrocontractile scarring can coexist. Retinal detachment and its repair can produce both photoreceptor injury and tractional fibrosis, including proliferative vitreoretinopathy (PVR), making the posterior segment a tractable setting in which to test potential antifibrotic-neuroprotective overlap (Townes-Anderson et al., 2017; Luyken et al., 2026).

In PVR, the evidence assembled here links ROCK signaling to retinal pigment epithelial (RPE) phenotypic remodeling and traction-producing cellular programs. Combined ROCK and TGF-β pathway inhibition reduced FN1 and α-SMA expression while preserving ZO-1, and a ROCK-inhibitor formulation attenuated experimental PVR (Chen et al., 2021; Ji et al., 2025). These data connect cytoskeletal regulation with epithelial-to-mesenchymal transition and fibrocontractile pathology. However, effects of combined inhibition cannot be assigned solely to ROCK, and experimental evidence do not yet establish translational relevance for the clinical prevention of PVR.

### 3.2 Other ocular fibrotic compartments and transcriptional mechanisms

ROCK-linked remodeling also occurs in ocular compartments outside PVR. In glucocorticoid-induced ocular hypertension, netarsudil improved outflow facility, reduced trabecular-meshwork stiffness, and decreased fibrosis-associated histologic readouts (Li et al., 2021). In CNV-associated subretinal fibrosis, fasudil and the ROCK2-selective inhibitor belumosudil reduced fibrotic remodeling and associated markers, including pMYPT1 (Li et al., 2025). These models illustrate how ROCK modulation may affect mechanically altered outflow tissue and posterior-segment scarring, although efficacy in one compartment does not establish efficacy in another.

Direct evidence for the transcriptional connection comes from ocular fibroblast studies linking mechanical strain and TGF-β-associated responses to RhoA/ROCK-dependent MRTF signaling (Yuan et al., 2018; Xie et al., 2022). In pterygium fibroblasts, ROCK inhibition blocked MRTF-A nuclear translocation and suppressed an SRF-associated program (Xie et al., 2022). This provides an ocular example of cytoskeletal regulation coupled to fibrogenic transcription. Its applicability to RPE, Müller glia, and other retinal cell states remains a question for direct testing.

### 3.3 The retina as an interface between scarring and neural injury

The retina is especially informative because structural remodeling and neuronal injury can occur in the same anatomical setting. Fasudil stabilized rod photoreceptor synapses and reduced outer-plexiform-layer injury in a retinal detachment model (Townes-Anderson et al., 2017). Separate optic-nerve and retinal studies found increased retinal ganglion cell survival and axon regeneration after topical AR-13324, and axonal protection after intravitreal netarsudil in a TNF-induced degeneration model (Shaw et al., 2017; Kitaoka et al., 2022). Together with the ocular fibrosis studies, these findings motivate testing antifibrotic and neuroprotective effects within the same disease model. Currently, there is no evidence that a single treatment improves both outcomes simultaneously in patients.

## 4 ROCK signaling in neurodegeneration and CNS injury

### 4.1 ROCK signaling in axonal regeneration

In the adult CNS, RhoA-ROCK signaling regulates cytoskeletal dynamics, neuronal morphology, and neuroimmune responses (Barcelo et al., 2023; Benarroch, 2023; Guan et al., 2023). Persistent pathway activation after injury or during neurodegeneration can contribute to regenerative failure, synaptic dysfunction, and maladaptive glial activation (Fu et al., 2007; Fujita and Yamashita, 2014).

Axonal regeneration is one of the best-established neural functions of the pathway. Following CNS injury, myelin-associated inhibitors such as Nogo-A, myelin-associated glycoprotein (MAG), and oligodendrocyte myelin glycoprotein engage RhoA-ROCK signaling, with downstream effects on LIM kinase and myosin light chain (Relton and Weinreb, 2008; Mehta et al., 2010; Hanifa et al., 2023; Nheu and Petratos, 2024). These cytoskeletal responses promote actomyosin contraction, growth-cone collapse, and axonal retraction (GrandPré et al., 2000; Wang et al., 2002; Fournier et al., 2003). ROCK inhibition can counter this inhibitory signaling and promote regeneration across CNS injury models (Roy et al., 2021), in part by reducing actin contraction and permitting microtubule advance within growth cones (Grandy et al., 2022). This mechanistic relationship places ROCK at a convergence point between extracellular growth-inhibitory cues and the intrinsic cytoskeletal machinery required for axonal extension.

### 4.2 ROCK regulates synaptic structure and plasticity

ROCK also regulates synaptic plasticity through the actin-rich cytoskeleton of dendritic spines (Benarroch, 2023). Dendritic spine stability and shape are major determinants of synaptic strength and plasticity (Hotulainen and Hoogenraad, 2010). RhoA-ROCK signaling influences actin turnover through LIM kinase and cofilin (Tsui et al., 2017), and experimental ROCK activation has been associated with spine shrinkage and synaptic destabilization, whereas inhibition favors spine enlargement and stabilization (Mulherkar et al., 2017). ROCK activity has also been linked to reduced long-term potentiation, a cellular correlate of learning and memory (Greathouse et al., 2018), while inhibition can enhance synaptic connectivity and functional recovery after CNS injury (Mulherkar et al., 2017). These findings extend the role of ROCK beyond growth-cone dynamics to the structural plasticity of established neural circuits.

### 4.3 ROCK signaling drives microglial activation and neuroinflammation

ROCK signaling also shapes neuroinflammatory responses (Roser et al., 2017; Lu et al., 2023). Microglial migration, phagocytosis, and cytokine release depend on dynamic cytoskeletal remodeling (Socodato and Relvas, 2024), and increased ROCK activity can promote an amoeboid, pro-inflammatory phenotype together with production of inflammatory cytokines and reactive oxygen species (Glotfelty et al., 2023). In experimental models, ROCK inhibitors reduce microglial activation and inflammatory mediator production (Scheiblich and Bicker, 2017; Glotfelty et al., 2023) and alter migration and phagocytic activity through effects on actomyosin contractility (Scheiblich and Bicker, 2017). Thus, the neuroprotective consequences of ROCK inhibition may reflect combined neuron-intrinsic effects on regeneration and synaptic structure together with modulation of the surrounding neuroimmune environment.

### 4.4 CNS scarring and the limits of an antifibrotic analogy

CNS injury-associated scars contain biologically distinct components that should not be grouped under a single antifibrotic mechanism. In a mouse spinal cord injury model, moderate reduction of pericyte-derived fibrotic scarring enhanced descending axonal regeneration and improved sensorimotor recovery (Dias et al., 2018). Conversely, preventing or ablating astrocytic scars did not permit spontaneous axon regrowth in severe spinal cord injury, and astrocytic scar formation supported experimentally stimulated regeneration (Anderson et al., 2016). These studies establish why scar composition and repair stage matter; neither directly demonstrates ROCK-dependent stromal fibrosis or an antifibrotic effect of ROCK inhibition in the CNS.

Downstream mechanotransduction studies reinforce this distinction. Astrocytic YAP activation promoted organized glial-scar formation, axonal regeneration, and functional recovery after experimental spinal cord injury, whereas YAP deletion impaired these responses (Xie et al., 2020). Such findings argue against indiscriminate suppression of reparative glial programs. A key unresolved question is whether ROCK can be modulated in stromal or inflammatory populations while preserving beneficial astrocytic functions. Testing this requires cell-specific perturbation and separate measurements of fibrotic matrix deposition, glial organization, and neural recovery.

### 4.5 Disease-specific evidence and context-dependent downstream responses

The mechanistic consequences of ROCK modulation also vary across degenerative disease models. In Friedreich ataxia, ROCK1/2 were identified as repressors of FXN, and pharmacological inhibition with agents including fasudil and belumosudil increased frataxin expression and improved mitochondrial and *in vivo* phenotypes (Fang et al., 2025). This illustrates a disease mechanism extending beyond fibrocontractile tension. In ALS, the phase 2 ROCK-ALS trial established the tolerability and feasibility of intravenous fasudil (Koch et al., 2024); this translational milestone should not be equated with established disease-modifying efficacy.

Evidence involving downstream transcriptional modules likewise cautions against assuming that ROCK inhibition and downstream pathway inhibition are interchangeable. YAP activation reduced amyloid-β-induced inflammatory signaling in microglial cells (Qing et al., 2020), and MRTF-A overexpression attenuated amyloid-β-associated neuronal injury and cognitive deficits in Alzheimer disease models (Zhang et al., 2022). Neuronal SRF promoted axonal regeneration and delayed disease progression in an ALS model (Stern et al., 2013; Song et al., 2023), whereas the small molecule MRTF/SRF inhibitor CCG-1423 reduced axonal elongation and dendritic complexity in cortical neurons (Kikuchi et al., 2017). These findings support context-specific investigation of downstream responses, rather than a uniform strategy of suppressing ROCK, YAP/TAZ, and MRTF/SRF together.

Across these settings, ROCK modulation can affect regeneration, synaptic organization, inflammatory responses, and ocular fibrocontractile remodeling through different cellular mechanisms (Figure 2). The central translational hypothesis is that selected interventions may influence more than one pathological compartment. Demonstrating this will require combined functional and structural endpoints, with isoform- and cell-specific measures of target engagement.

**FIGURE 2:**
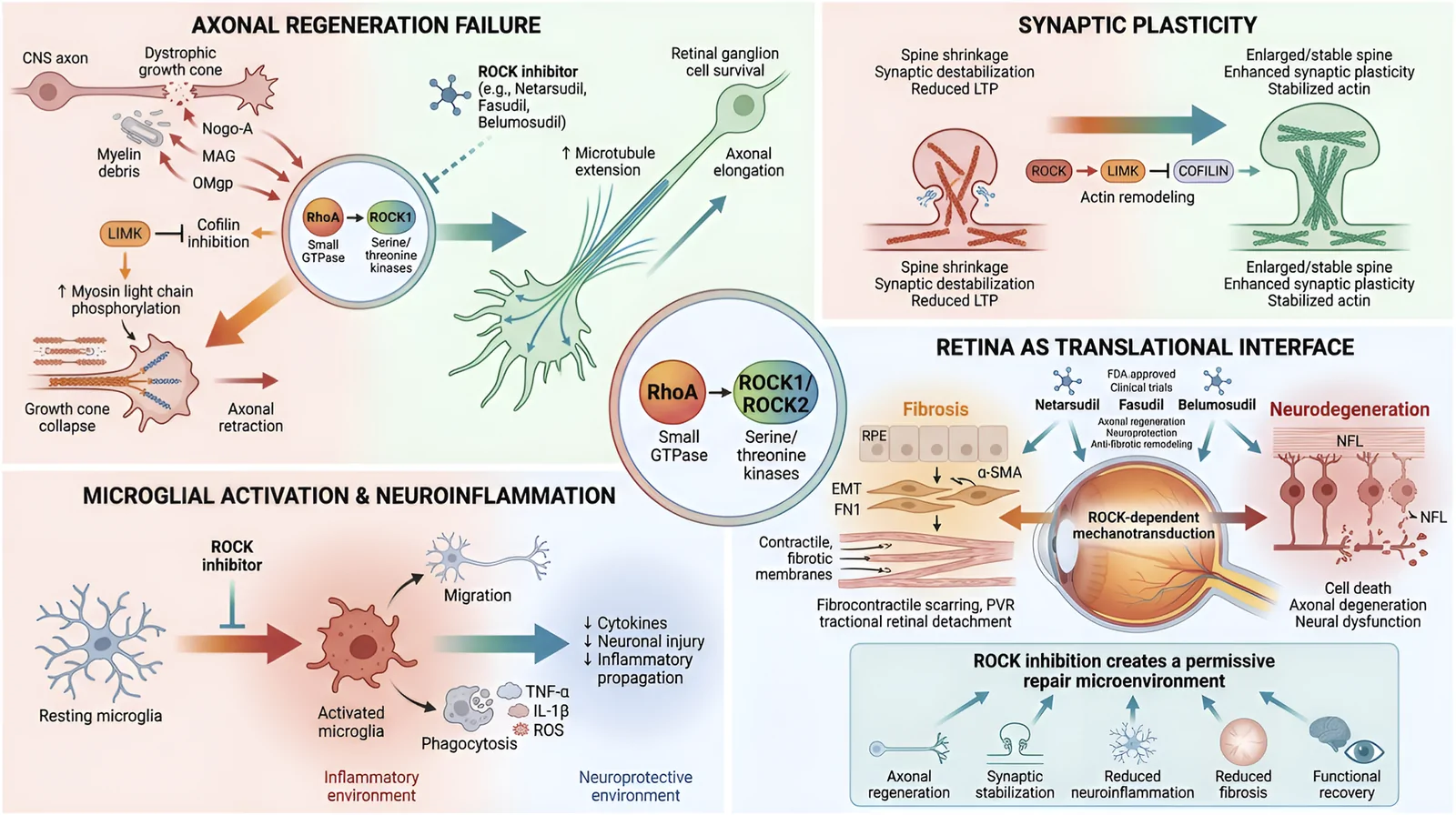
ROCK signaling as a convergent regulator of neurodegeneration, neuroinflammation, regeneration failure, and fibrocontractile remodeling. This summarizes ROCK-dependent mechanisms that connect neuronal injury, neuroinflammation, synaptic dysfunction, and retinal fibrosis. In axonal regeneration failure, myelin-associated inhibitory cues, including Nogo-A, MAG, and OMgp, activate RhoA–ROCK signaling, leading to LIMK activation, cofilin inhibition, increased myosin light chain phosphorylation, growth cone collapse, and axonal retraction. ROCK inhibition with clinically relevant agents such as netarsudil, fasudil, or belumosudil can promote microtubule extension, axonal elongation, and retinal ganglion cell survival. In synaptic plasticity, ROCK regulation of the LIMK–cofilin axis controls actin remodeling and dendritic spine stability, with ROCK inhibition favoring enlarged, stable spines and enhanced synaptic plasticity. ROCK signaling also contributes to microglial activation, migration, phagocytosis, and inflammatory mediator production, including TNF-α, IL-1β, and ROS; inhibition shifts this response toward a more neuroprotective environment. The retina is highlighted as a translational interface where ROCK-dependent mechanotransduction contributes both to fibrocontractile scarring, including RPE EMT, FN1 accumulation, α-SMA induction, PVR membrane contraction, tractional retinal detachment, and to neurodegenerative outcomes such as nerve fiber layer loss, axonal degeneration, and neural dysfunction. Together, the figure illustrates how ROCK inhibition may create a permissive repair microenvironment by promoting axonal regeneration, synaptic stabilization, reduced neuroinflammation, reduced fibrosis, and functional recovery.

## 5 Context-dependent ROCK1 and ROCK2 functions in ocular and neural disease

ROCK1 and ROCK2 have overlapping functions in cytoskeletal remodeling, contractility, and motility, but isoform-associated outcomes depend on tissue and cell state. Neural studies implicate both isoforms in synaptic function and amyloid-associated phenotypes (Chaturvedi et al., 2011; Henderson et al., 2016; Yan et al., 2019; Zhao et al., 2020), and isoform-specific transcriptomic analyses show overlapping but non-identical coding and long noncoding RNA programs (Zhou et al., 2019). These observations support context-dependent specialization rather than a universal ROCK1-versus-ROCK2 division of labor. The broader substrate catalogue is included in Supplementary Table S1; it distinguishes shared regulation from preferential isoform-associated evidence. Figure 3 summarizes the shared signaling core and context-dependent specialization of ROCK1 and ROCK2. Evidence from peripheral systems provides comparative context, while the ocular and CNS examples highlight isoform-associated responses relevant to this review.

**FIGURE 3:**
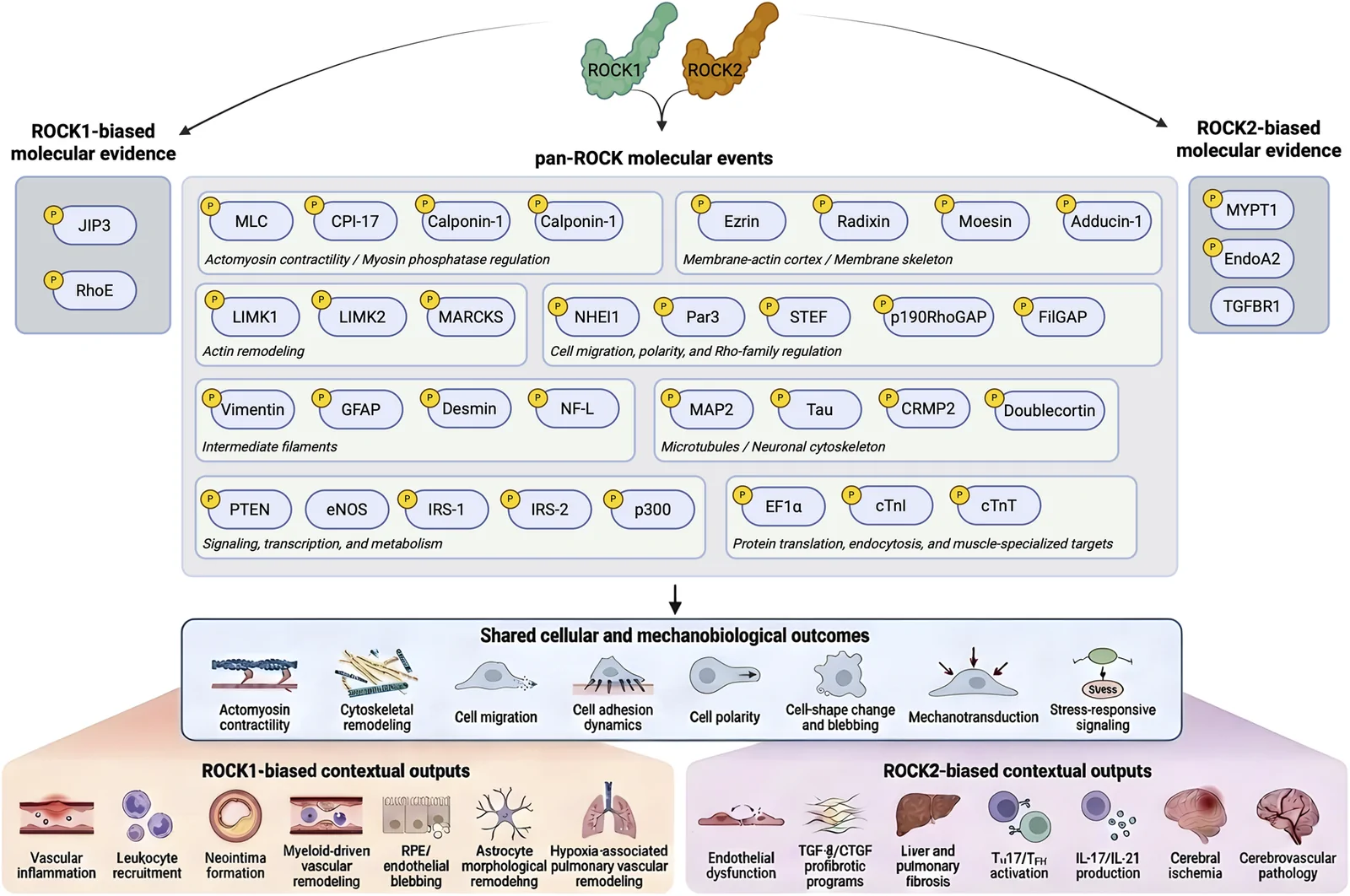
Context-dependent ROCK1 and ROCK2 specialization provides a framework for interpreting ocular and CNS signaling. ROCK1 and ROCK2 share a conserved core of cytoskeletal regulation while displaying context-dependent differences in molecular interactions and biological outcomes. The central module summarizes ROCK-regulated targets involved in actomyosin contractility, actin remodeling, adhesion, polarity, and stress-responsive signaling. Isoform-biased molecular relationships and tissue-level responses illustrate partial specialization rather than exclusive substrate specificity or a universal division of labor. Ocular and CNS examples include RPE/endothelial blebbing, astrocyte remodeling, cerebral ischemia, and cerebrovascular pathology. Peripheral vascular, immune, and fibrotic examples illustrate the context-dependent roles of ROCK1 and ROCK2 across cell types and tissue microenvironments. Isoform-associated responses shown in these tissues are not necessarily conserved in ocular or CNS populations.

Ocular and neural examples are particularly relevant to this distinction. ROCK1-associated studies include diabetes-related RPE and endothelial blebbing and astrocyte morphological changes after epileptic activity (Vemula et al., 2010; Rothschild et al., 2017; Anders et al., 2024). Selective ROCK2 inhibition has shown benefit in focal cerebral ischemia and cavernous angioma models (Lee et al., 2014a; McKerracher et al., 2020), and belumosudil reduced fibrosis in a CNV-associated subretinal fibrosis model (Li et al., 2025). These findings identify testable isoform biases, but they do not establish that ROCK1 is exclusively cytoskeletal or ROCK2 exclusively inflammatory or fibrotic.

A further limitation is that preferential biological outcomes do not establish exclusive kinase-substrate relationships (Mertsch and Thanos, 2014; Zhou et al., 2019). Many pharmacological studies also do not resolve ROCK1 and ROCK2 contributions in each responding population. Direct comparisons of dual and isoform-selective inhibition should therefore be paired with genetic perturbation, phosphorylation-based target-engagement measurements, and outcomes relevant to neurons, glia, vascular cells, and ocular fibrotic populations.

## 6 Cell-state and multi-omics approaches in ocular and neural disease

### 6.1 The limits of bulk approaches and the need for cell-state resolution

ROCK signaling is strongly shaped by cellular context, yet much of the available evidence derives from bulk tissue or population-averaged measurements. ROCK1 and ROCK2 regulate overlapping but non-identical transcriptional programs (Hartmann et al., 2015; Zhou et al., 2019), and bulk analyses can obscure whether an apparent pathway change reflects altered signaling within a cell population, a shift in cellular composition, or both. This limitation is particularly important in fibrotic and neural tissues, where fibroblasts, immune cells, vascular cells, glia, and neuronal subtypes occupy distinct states and may differ substantially in their dependence on ROCK. Single-cell and spatial approaches therefore offer a way to localize ROCK-associated programs to defined cell populations rather than treating tissue-level expression as a uniform pathway readout.

Transcript abundance alone is insufficient to establish ROCK activity. As a serine/threonine kinase, ROCK regulates phosphorylation-dependent cytoskeletal responses involving substrates such as MLC, MYPT1, and LIMK; downstream readouts can include cofilin phosphorylation (Hartmann et al., 2015). Protein-level responses to ROCK inhibition need not be mirrored by corresponding mRNA changes (Gharechahi et al., 2014). Integrating single-cell and spatial transcriptomics with chromatin-accessibility profiling, proteomics, and phosphoproteomics can therefore connect cellular identity and transcriptional state to more direct measures of pathway output. Figure 4 illustrates this methodological framework, including broader disease applications that provide comparative context. Here, its principal relevance is to resolving ROCK-associated states in ocular and CNS populations and linking those states to functional pathway dependence.

**FIGURE 4:**
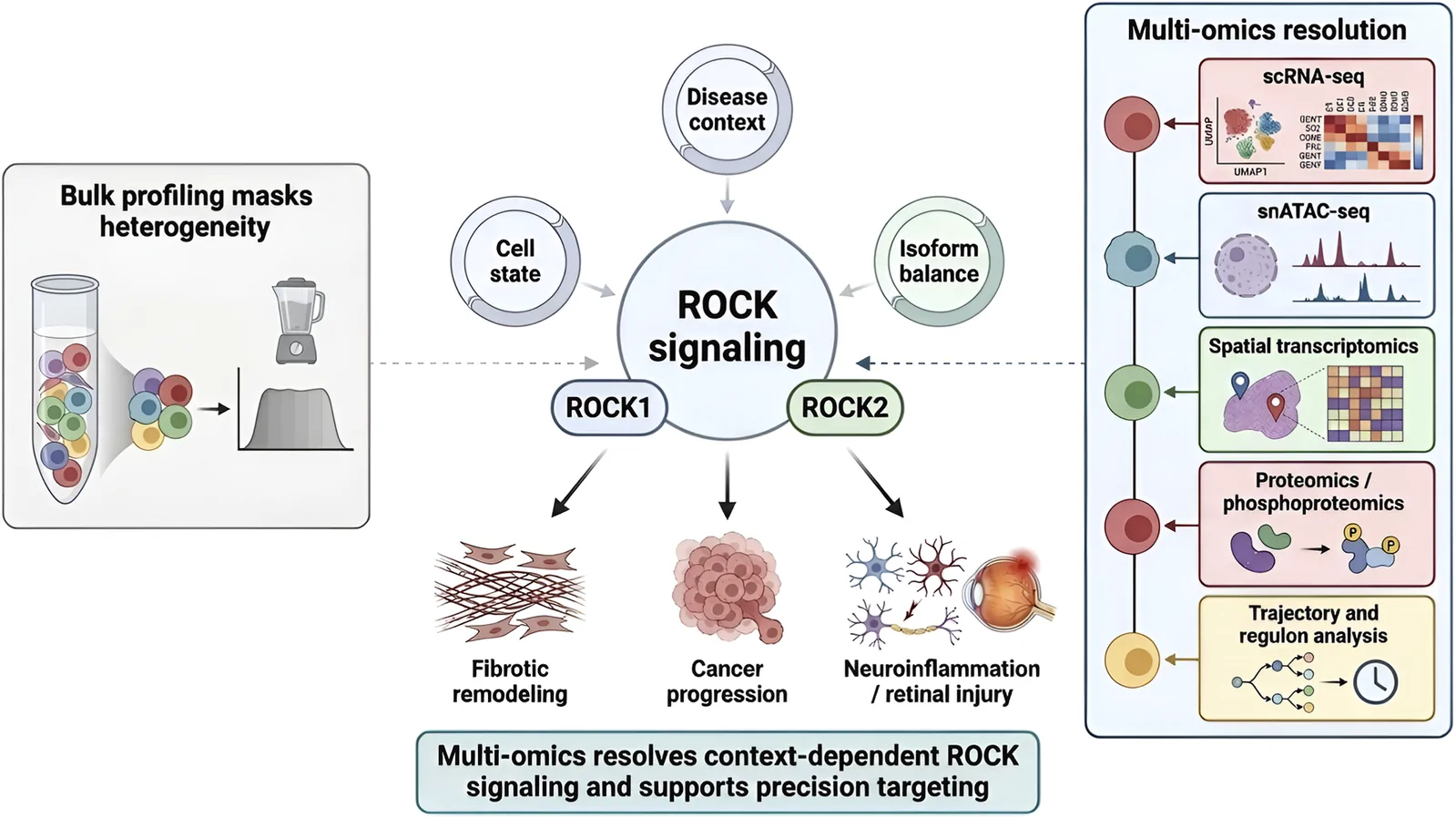
Single-cell and multi-omics approaches provide a framework for resolving ROCK-associated cell states in ocular and CNS disease. Bulk profiling averages signals across heterogeneous populations and may obscure cell-specific responses or changes in tissue composition. Single-cell RNA sequencing, single-nucleus chromatin-accessibility profiling, spatial transcriptomics, proteomics/phosphoproteomics, and trajectory or regulon analyses provide complementary information about cellular identity, regulatory state, spatial organization, and signaling output. In ocular and CNS disease, these approaches can help characterize ROCK-associated programs in neuronal, glial, immune, vascular, and fibrotic populations, including those involved in retinal injury and neuroinflammation. Fibrotic remodeling and cancer progression are shown as broader comparative applications of the methodological framework. Transcript abundance, inferred regulatory activity, and computational trajectories alone do not establish ROCK activation or isoform dependence. Linking these measurements to selective perturbation and phosphorylation-based target-engagement assays is necessary to identify causal ROCK-dependent programs and evaluate their therapeutic relevance.

### 6.2 Retinal cell populations and disease-associated states

The retina is particularly well suited to extending this framework into a neural tissue because its cellular composition and neuronal diversity have been mapped at single-cell resolution. Human retinal and murine retinal ganglion cell atlases provide reference frameworks for examining ROCK1 and ROCK2 expression across defined cell types (Rheaume et al., 2018; Lukowski et al., 2019), and retinal ganglion cell subtypes show marked differences in transcriptional resilience after injury (Tran et al., 2019). More directly, single-cell analysis after optic nerve injury identified a GPR84-expressing microglial population that promotes TNF/IL-1alpha signaling through the Rho-ROCK pathway (Sato et al., 2023). This provides an example of how cell-state-resolved profiling can localize ROCK-associated pathology to a specific neural immune population. Retinal atlases primarily establish cell identity and expression patterns; they do not by themselves demonstrate ROCK activity, isoform dependence, or therapeutic accessibility.

Ocular fibroblast experiments provide a complementary perturbation-based anchor: ROCK inhibition alters MRTF-A nuclear localization and SRF-associated transcription in pterygium fibroblasts (Xie et al., 2022). Combining this type of functional evidence with retinal or CNS cell atlases could identify which populations engage related programs after injury. Trajectory, RNA-velocity, and regulon analyses can help characterize state transitions (Bergen et al., 2020; Qiu et al., 2022; Weiler et al., 2024), but computationally inferred transitions or YAP/TEAD and MRTF/SRF signatures cannot independently establish ROCK dependence.

### 6.3 Experimental priorities and therapeutic implications

The principal experimental gap is therefore not the absence of single-cell technologies, but the scarcity of perturbation-resolved datasets designed specifically around ROCK biology. Future studies should combine isoform-selective genetic or pharmacological perturbation with scRNA-seq, spatial transcriptomics, chromatin-accessibility profiling, and phosphoproteomics in the same disease models. Longitudinal or trajectory-aware designs would be particularly valuable for distinguishing transient adaptive responses from stable pathological cell states and for identifying when ROCK1- or ROCK2-dependent programs become therapeutically actionable. In ocular and neural systems, where direct isoform-resolved evidence remains comparatively limited, these approaches should prioritize disease-relevant glial, neuronal, vascular, immune, and fibrotic populations rather than infer pathway behavior solely from bulk tissue or from non-neural models.

Such datasets would also provide a stronger basis for translation. Cell-state-resolved pathway activity could help distinguish settings in which pan-ROCK inhibition is appropriate from those in which isoform-selective or spatially restricted modulation is more likely to preserve physiological signaling while suppressing pathological remodeling. Integrating transcriptional state with direct measurements of kinase output is therefore essential for moving from descriptive ROCK expression maps toward a causal, cell-state-specific model of signaling. This transition is particularly important for the eye and CNS, where therapeutic precision will depend on defining not only whether ROCK is present, but which isoform is active, in which cell population, at what disease stage, and with what downstream consequence.

## 7 Therapeutics and future directions

### 7.1 Delivery strategies for spatially restricted ROCK modulation

Therapeutic precision is a central challenge because systemic non-selective ROCK inhibition can produce dose-limiting effects, including hypotension (Barcelo et al., 2023; Sambandam et al., 2023), while local ocular treatment can cause adverse effects such as conjunctival hyperemia and blepharitis (Chatzimichail et al., 2025). Spatially restricted delivery, molecular selectivity, and intervention timing should therefore be evaluated together. The relevant goal in eye and brain disease is sufficient modulation of a pathogenic population while preserving physiological repair and limiting exposure in other compartments (Figure 5).

**FIGURE 5:**
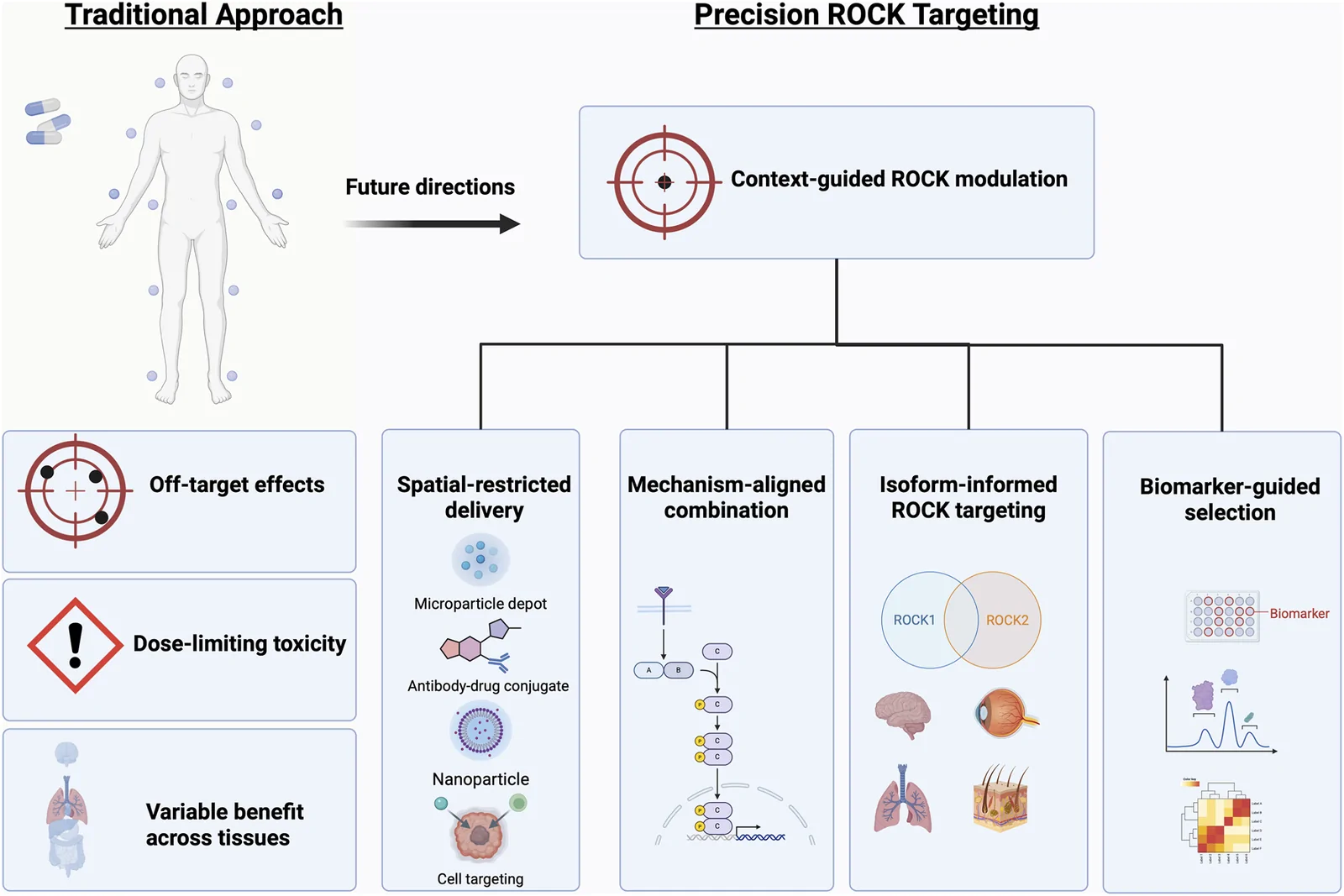
Precision ROCK targeting as a future direction for ROCK-based therapeutics. This schematic contrasts traditional systemic ROCK inhibition with a precision ROCK-targeting framework designed to improve therapeutic efficacy and tolerability. Broad ROCK modulation can produce beneficial effects in selected disease settings but may be limited by off-target activity, dose-limiting toxicity, and variable responses across tissues. Future strategies should therefore emphasize context-guided ROCK modulation through spatially restricted delivery, mechanism-aligned combination therapy, isoform-informed ROCK1/ROCK2 targeting, and biomarker-guided patient or tissue selection. Together, these approaches may enable more selective inhibition of disease-relevant ROCK signaling while reducing systemic exposure and minimizing suppression of ROCK functions that are beneficial or context dependent.

Several delivery strategies illustrate this principle. Nanoparticle formulations of fasudil can improve ocular permeability and sustain drug release (Osi et al., 2024), while CD31 antibody-conjugated nanoparticles provide proof-of-concept for endothelial-restricted RhoA/ROCK inhibition (Lahooti et al., 2023). Depot formulations may prolong intraocular exposure (Delong et al., 2020; Tsujinaka et al., 2020), and suprachoroidal administration offers direct access to posterior ocular tissues with reduced systemic exposure (Wu et al., 2024). Stimuli-responsive nano-micro systems could further restrict release according to local features such as pH, reactive oxygen species, or mechanical stress (Ahmed et al., 2023), an attractive concept for mechanically abnormal regions in which ROCK-linked signaling is enriched (Hadzipasic et al., 2025; Min et al., 2025).

Importantly, these approaches are at different stages of validation. Some have been tested directly with ROCK inhibitors, whereas others remain enabling delivery platforms whose value for ROCK-directed therapy is still prospective. The translational priority is therefore to establish whether spatially restricted delivery improves target engagement and therapeutic index in disease-relevant tissues, particularly in ocular and neural settings where chronic systemic inhibition may be undesirable.

### 7.2 Posterior ocular disease and clinical translation

Netarsudil is a clinically established ophthalmic ROCK/NET inhibitor (Karri and Chong, 2023), but posterior-segment efficacy requires additional exposure and target-engagement evidence. Rabbit topical-dosing studies detected drug-related radioactivity in retina-choroid and vitreous without establishing unbound human vitreous concentrations (U.S. Food and Drug Administration, 2018). The trial records cited in this review describe evaluation of topical netarsudil for PVR prevention and of posterior-segment safety and pharmacokinetics (Clinicaltrials.Gov:Nct05660447, 2022; Clinicaltrials.Gov:Nct06033703, 2023).

Beyond topical treatment, systemic ROCK inhibition has also been evaluated in diabetic retinal disease. The phase 2 SPECTRA trial investigated oral OPL-0401, a dual ROCK1/2 inhibitor, in patients with diabetic retinopathy with or without diabetic macular edema (DME) (Clinicaltrials.Gov:Nct05393284, 2022). In this randomized, double-masked, placebo-controlled study, 114 patients received treatment over 24 weeks. Sponsor-reported topline results indicated that the primary and key secondary efficacy endpoints were not met, despite favorable tolerability; exploratory signals suggesting reduced disease progression at certain doses require confirmation (Valo HealthInc, 2024). These findings extend the clinical evaluation of ROCK inhibition to posterior retinal disease while underscoring the need to establish efficacy for each indication and endpoint.

### 7.3 Mechanism-aligned combination strategies

Combination therapy offers a complementary route to therapeutic precision. Because ROCK regulates cytoskeletal tension and mechanotransduction rather than every component of complex disease biology, pairing ROCK inhibition with agents that address parallel pathogenic programs may increase therapeutic coverage (Sun et al., 2016). The rationale is strongest when the combination is mechanism-aligned rather than empiric, with each intervention addressing a distinct but interacting disease process.

Clinical evidence for this strategy comes from the phase 3 MERCURY-1 and MERCURY-2 trials, in which a fixed-dose combination of netarsudil and latanoprost produced greater intraocular pressure reduction than either component alone in patients with open-angle glaucoma or ocular hypertension (Asrani et al., 2020). Other ocular examples include combinations with prostaglandin analogs (Lee et al., 2022), anti-VEGF therapy (Fekri et al., 2022), and YAP-directed approaches (Zhang et al., 2025). Their interpretation must remain tied to the tested indication and model: effects on pressure, vascular leakage, or corneal repair do not establish effects on retinal fibrosis or neuronal survival. ROCK-associated lysosomal biology also suggests potential combinations in neurodegeneration (Song et al., 2024; Zheng et al., 2025), although direct evidence of additive benefit remains limited. Downstream inhibition should be approached particularly cautiously where YAP/TAZ or MRTF/SRF support beneficial neuronal or glial responses.

### 7.4 Future directions: isoform-selective and context-matched ROCK targeting

Clinical experience with fasudil in neurovascular settings (Masaoka et al., 2001; Koch et al., 2018) and with ripasudil and netarsudil in the eye (Tanihara et al., 2015; Inazaki et al., 2017; Tanna and Johnson, 2018; Araie et al., 2023) establishes pharmacological tractability. In glaucoma and ocular hypertension, the phase 3 ROCKET-1 and ROCKET-2 trials evaluated netarsudil against timolol and demonstrated clinically meaningful intraocular pressure reduction (Serle et al., 2018), while J-ROCKET directly compared netarsudil with ripasudil (Araie et al., 2023). These trials establish ocular hypotensive efficacy, while direct neuroprotective and antifibrotic effects require separate clinical evaluation. Belumosudil provides a comparative example of sustained ROCK2-preferring inhibition in humans (Cutler et al., 2021; Przepiorka et al., 2022), while its ocular and neurodegenerative applications remain supported here chiefly by experimental models. The priority is to determine when isoform selectivity improves outcome in a particular ocular or neural disease, rather than infer benefit from systemic indications.

The next step is not simply to generate more selective compounds, but to determine when selectivity improves outcome. Direct comparisons of pan-ROCK and isoform-selective inhibition should be performed in disease-relevant cell states and tissues, with pharmacodynamic measures of target engagement and endpoints that capture both efficacy and preservation of physiological function. Coupling molecular selectivity with spatially restricted delivery may ultimately provide greater precision than either strategy alone.

Future studies should test whether combined antifibrotic and neuroprotective benefit occurs within the same ocular or CNS model. Endpoints should separately capture matrix accumulation and contraction, neuronal survival and function, glial responses, and vascular integrity. Linking these outcomes to local drug exposure, substrate phosphorylation, and isoform-specific perturbation would distinguish mechanistic benefit from general pathway association. Such designs should also identify treatment windows in which pathological remodeling can be reduced without disrupting protective scar organization or tissue repair.

## 8 Conclusion

ROCK signaling provides a mechanistic interface between ocular fibrocontractile remodeling and neural dysfunction through its regulation of cytoskeletal organization, contractility, and cellular responses to injury. The retina is a particularly informative setting because fibrotic scarring and neuronal damage can coexist. However, evidence from separate antifibrotic and neuroprotective studies does not establish simultaneous clinical benefit, and CNS stromal fibrosis must be distinguished from astrocytic scarring that can support repair. The central implication is that therapeutic value depends on the responding cell population, anatomical compartment, and disease stage, rather than on uniform pathway suppression.

Future studies should define ROCK1 and ROCK2 requirements in disease-relevant ocular and neural populations using selective perturbation and phosphorylation-based measures of target engagement. Single-cell and spatial approaches can guide these experiments, but expression maps and downstream transcriptional signatures require causal validation. Local delivery and mechanism-aligned combinations should be tested against outcomes that separately quantify fibrosis, neural survival and function, and preservation of physiological repair. Resolving when and where ROCK modulation achieves these objectives will determine whether the shared mechanistic framework can support effective treatment of ocular fibrosis and neurodegenerative disease.
