# Supplementary material for: ROCK signaling at the interface of fibrosis and neurodegeneration: mechanisms and therapeutic potential
Source: Front Cell Dev Biol. 2026 Oct 30;14:1933409. doi: 10.3389/fcell.2026.1933409 (PMC13759834; doi:10.3389/fcell.2026.1933409)
Supplement: Supplementary file 1 [file Supplementaryfile1.pdf]

ROCK Signaling at the Interface of Fibrosis and Neurodegeneration: Mechanisms and Therapeutic Potential

Jeysson Sanchez-Suarez[1,2], Yvonne Adu-Rutledge[1,2,†], Kunho Bae[1,2,3,†], Hyungwoo Lee[1,2,4†], Anton Lennikov[1,2,5,†], Nakyung Ryoo[1,2,†], Anil Upreti[1,2,†], Aruvi Vijikumar[1,2,†], William P. Miller[1,2,*], Leo A. Kim[1,2,*]

[1]Schepens Eye Research Institute of Massachusetts Eye and Ear Infirmary, Boston, MA, United States.
[2] Department of Ophthalmology, Harvard Medical School; Boston, MA, United States.
[3]Department of Ophthalmology, Biomedical Research Institute, Seoul National University Hospital; Seoul, Korea
[4]Department of Ophthalmology, Konkuk University School of Medicine, Konkuk University Medical Center, Seoul, Republic of Korea.
[5]Department of Ophthalmology and Visual Neuroscience, University of Minnesota, Minneapolis, Minnesota, United States.

Supplementary Table S1. Experimentally reported ROCK substrates and interacting targets with current evidence for ROCK1- and ROCK2-specific regulation.

**Target**	**Evidence for ROCK1**	**Evidence for ROCK2**	**Main function[a]**	**Refs.**
**MYPT1**	Shared / pan-ROCK; no clear direct ROCK1 assignment	Direct binding/phosphorylation explicitly assigned to ROCK2	Regulatory subunit of myosin phosphatase that targets the phosphatase to myosin and helps control myosin dephosphorylation.	(Kimura et al., 1996;Amano et al., 2010a;Hahmann and Schroeter, 2010;Surma et al., 2011)
**MLC2**	pan-ROCK	pan-ROCK	Regulatory myosin light chain that controls smooth-muscle and nonmuscle contractility through phosphorylation.	(Amano et al., 1996;Amano et al., 2010a;Hahmann and Schroeter, 2010;Surma et al., 2011)

Target	Evidence for ROCK1	Evidence for ROCK2	Main function[a]	Refs.
**Ezrin**	pan-ROCK	pan-ROCK	Membrane–cytoskeleton linker that connects cortical actin structures to the plasma membrane.	(Matsui et al., 1998;Amano et al., 2010a;Hahmann and Schroeter, 2010;Surma et al., 2011)
**Radixin**	pan-ROCK	pan-ROCK	Membrane–cytoskeleton linker that helps connect actin filaments to the plasma membrane.	(Matsui et al., 1998;Amano et al., 2010a;Hahmann and Schroeter, 2010;Surma et al., 2011)
**Moesin**	pan-ROCK	pan-ROCK	ERM-family membrane–cytoskeleton linker important for cell cortex organization.	(Matsui et al., 1998;Amano et al., 2010a;Hahmann and Schroeter, 2010;Surma et al., 2011)

Target	Evidence for ROCK1	Evidence for ROCK2	Main function[a]	Refs.
**α-Adducin**	pan-ROCK	pan-ROCK	Membrane skeleton protein that promotes assembly of the spectrin–actin network.	(Fukata et al., 1999;Amano et al., 2010a;Hahmann and Schroeter, 2010;Surma et al., 2011)
**LIMK1**	pan-ROCK	pan-ROCK	Serine/threonine kinase that regulates actin dynamics and actin-dependent processes such as motility and differentiation.	(Ohashi et al., 2000;Amano et al., 2010a;Hahmann and Schroeter, 2010;Surma et al., 2011)
**LIMK2**	Shared / pan-ROCK; ROCK1–LIMK2 interaction specifically noted	pan-ROCK	Serine/threonine kinase with an essential role in regulation of actin filament dynamics.	(Sumi et al., 2001;Hahmann and Schroeter, 2010;Surma et al., 2011)
**Calponin-1**	Pan-ROCK	Pan-ROCK	Thin-filament-associated protein involved in regulation and modulation of smooth-muscle contraction.	(Kaneko et al., 2000;Amano et al., 2010a)

**Target**	**Evidence for ROCK1**	**Evidence for ROCK2**	**Main function[a]**	**Refs.**
**CPI-17**	Pan-ROCK	Pan-ROCK	Protein phosphatase inhibitor that becomes a potent inhibitor of myosin phosphatase when phosphorylated.	(Koyama et al., 2000;Amano et al., 2010a;Nunes et al., 2010)
**Na^+/H^+ exchanger 1**	Pan-ROCK	Pan-ROCK	Na+/H+ exchanger that regulates intracellular pH and cell volume and supports growth, proliferation, migration, and survival.	(Amano et al., 2010a;Dong et al., 2010;Wallert et al., 2015)
**MARCKS**	Pan-ROCK	Pan-ROCK	Membrane-associated protein involved in structural modulation of the actin cytoskeleton, chemotaxis, motility, cell adhesion, and phagocytosis.	(Nagumo et al., 2001;Amano et al., 2010a)
**eEF1α**	Pan-ROCK	Pan-ROCK	Translation elongation factor that catalyzes the GTP-dependent delivery of aminoacyl-tRNA to the ribosomal A-site during elongation.	(Amano et al., 2010a;Hiroi et al., 2018)
**Cardiac troponin I**	Pan-ROCK	Pan-ROCK	Inhibitory subunit of troponin, the thin-filament regulatory complex that confers calcium sensitivity to striated muscle actomyosin ATPase activity.	(Vahebi et al., 2005;Amano et al., 2010a)

Target	Evidence for ROCK1	Evidence for ROCK2	Main function[a]	Refs.
**Cardiac troponin T**	Pan-ROCK	Pan-ROCK	Tropomyosin-binding subunit of troponin, the thin-filament regulatory complex that confers calcium sensitivity to striated muscle actomyosin ATPase activity.	(Vahebi et al., 2005;Amano et al., 2010a)
**MAP2**	Pan-ROCK	Pan-ROCK	Microtubule-associated protein; MAP family proteins help stabilize microtubules against depolymerization.	(Amano et al., 2003;Amano et al., 2010a)
**Tau**	Pan-ROCK	Pan-ROCK	Promotes microtubule assembly and stability and may help establish and maintain neuronal polarity.	(Amano et al., 2003;Amano et al., 2010a)
**CRMP2**	Pan-ROCK	Pan-ROCK	Plays a role in neuronal development and polarity, axon growth and guidance, growth cone collapse, and cell migration.	(Arimura et al., 2000;Amano et al., 2010a)
**Doublecortin**	Pan-ROCK	Pan-ROCK	Microtubule-associated protein required for neuronal migration and cortical layering.	(Amano et al., 2010a;Amano et al., 2010b)
**GFAP**	Pan-ROCK	Pan-ROCK	Class III intermediate filament protein and a cell-specific astrocyte marker during CNS development.	(Kosako et al., 1997;Amano et al., 2010a)

Target	Evidence for ROCK1	Evidence for ROCK2	Main function[a]	Refs.
**Vimentin**	Pan-ROCK	Pan-ROCK	Intermediate filament protein involved in directional cell movement, cell-sheet organization, and Golgi polarization during migration.	(Goto et al., 1998;Amano et al., 2010a;Dong et al., 2010)
**Desmin**	Pan-ROCK	Pan-ROCK	Muscle-specific type III intermediate filament essential for proper muscular structure and function.	(Inada et al., 1998;Dong et al., 2010)
**NF-L**	Pan-ROCK	Pan-ROCK	Intermediate filament component of neurofilaments involved in maintenance of neuronal caliber.	(Amano et al., 2010a;Kang et al., 2011)
**Par3**	Pan-ROCK	Pan-ROCK	Adapter protein involved in cell polarity and asymmetric cell division.	(Nakayama et al., 2008;Amano et al., 2010a)
**STEF**	Pan-ROCK	Pan-ROCK	GDP-dissociation stimulator protein that activates Rho-family GTPases by promoting GDP–GTP exchange.	(Takefuji et al., 2007;Amano et al., 2010a)
**p190RhoGAP**	Pan-ROCK	Pan-ROCK	Rho GTPase-activating protein involved in cell differentiation, adhesion, and migration.	(Mori et al., 2009;Amano et al., 2010a)

Target	Evidence for ROCK1	Evidence for ROCK2	Main function[a]	Refs.
**eNOS**	Pan-ROCK	Pan-ROCK	Produces nitric oxide, which promotes vascular smooth-muscle relaxation through cGMP signaling.	(Ming et al., 2002;Amano et al., 2010a;Dong et al., 2010)
**PTEN**	Pan-ROCK	Pan-ROCK	Lipid and protein phosphatase that antagonizes phosphoinositide signaling.	(Li et al., 2005;Amano et al., 2010a;Surma et al., 2011)
**FilGAP**	Pan-ROCK	Pan-ROCK	Rho GTPase-activating protein involved in cell polarity, morphology, and cytoskeletal organization; acts mainly on Rac-family GTPases.	(Ohta et al., 2006;Amano et al., 2010a)
**IRS1**	Pan-ROCK	Pan-ROCK	Signaling adaptor that propagates insulin signaling by recruiting SH2-domain-containing proteins.	(Furukawa et al., 2005;Lim et al., 2007;Amano et al., 2010a;Surma et al., 2011)
**IRS2**	Pan-ROCK	Pan-ROCK	Insulin receptor substrate family signaling adaptor.	(Lim et al., 2007;Surma et al., 2011)

Target	Evidence for ROCK1	Evidence for ROCK2	Main function[a]	Refs.
**Endophilin-A1**	pan-ROCK	Pan-ROCK	Endocytic protein implicated in endocytosis and recruitment of proteins to highly curved membranes.	(Kaneko et al., 2005;Amano et al., 2010a)
**p300**	Pan-ROCK	Pan-ROCK	Histone acetyltransferase and transcriptional coactivator that regulates transcription through chromatin remodeling and acetylation of histone and non-histone proteins.	(Tanaka et al., 2006;Amano et al., 2010a)
**RhoE**	Evidence favors ROCK1-related regulation	Not clearly supported	Atypical Rho-family GTP-binding protein that binds GTP but lacks intrinsic GTPase activity and is resistant to RhoGAPs.	(Riento et al., 2005;Amano et al., 2010a;Hahmann and Schroeter, 2010)
**JIP3**	Explicitly assigned to ROCK1	No explicit ROCK2 assignment	Scaffold/adaptor protein for JNK signaling and intracellular transport.	(Ongusaha et al., 2008;Hahmann and Schroeter, 2010)
**TGFBR1**	No explicit ROCK1 assignment	Explicitly assigned to ROCK2	Transmembrane serine/threonine kinase receptor for TGF-beta family ligands.	(Hahmann and Schroeter, 2010;Bai et al., 2026)

Targets are organized according to the strength of isoform assignment derived from the reviewed literature, distinguishing shared (pan-ROCK) regulation from preferential ROCK1- or ROCK2-associated evidence when available. The table highlights canonical cytoskeletal regulators (e.g., MYPT1, MLC, ERM proteins, LIMKs), neuronal and microtubule-associated proteins, polarity regulators, signaling adaptors, and transcriptional modulators. Functional annotations describe the principal biological role of each target rather than the specific ROCK-dependent effect. Importantly, for many targets, direct isoform specificity remains unresolved and current evidence supports shared ROCK regulation rather than definitive ROCK1- or ROCK2-exclusive signaling.

References

Amano, M., Ito, M., Kimura, K., Fukata, Y., Chihara, K., Nakano, T., Matsuura, Y., and Kaibuchi, K. (1996). Phosphorylation and activation of myosin by Rho-associated kinase (Rho-kinase). *J Biol Chem* 271**,** 20246-20249.

Amano, M., Kaneko, T., Maeda, A., Nakayama, M., Ito, M., Yamauchi, T., Goto, H., Fukata, Y., Oshiro, N., Shinohara, A., Iwamatsu, A., and Kaibuchi, K. (2003). Identification of Tau and MAP2 as novel substrates of Rho-kinase and myosin phosphatase. *J Neurochem* 87**,** 780-790.

Amano, M., Nakayama, M., and Kaibuchi, K. (2010a). Rho-kinase/ROCK: A key regulator of the cytoskeleton and cell polarity. *Cytoskeleton (Hoboken)* 67**,** 545-554.

Amano, M., Tsumura, Y., Taki, K., Harada, H., Mori, K., Nishioka, T., Kato, K., Suzuki, T., Nishioka, Y., Iwamatsu, A., and Kaibuchi, K. (2010b). A proteomic approach for comprehensively screening substrates of protein kinases such as Rho-kinase. *PLoS One* 5**,** e8704.

Arimura, N., Inagaki, N., Chihara, K., Menager, C., Nakamura, N., Amano, M., Iwamatsu, A., Goshima, Y., and Kaibuchi, K. (2000). Phosphorylation of collapsin response mediator protein-2 by Rho-kinase. Evidence for two separate signaling pathways for growth cone collapse. *J Biol Chem* 275**,** 23973-23980.

Bai, Y., Ali, M.M., Van Dinther, M., Ten Dijke, P., Moustakas, A., Sundqvist, A., and Heldin, C.H. (2026). Opposing effects of Rho-associated coiled-coil kinase 1 (ROCK1) and ROCK2 in TGF-beta-SMAD signaling. *Cell Commun Signal* 24.

Dong, M., Yan, B.P., Liao, J.K., Lam, Y.Y., Yip, G.W., and Yu, C.M. (2010). Rho-kinase inhibition: a novel therapeutic target for the treatment of cardiovascular diseases. *Drug Discov Today* 15**,** 622-629.

Fukata, Y., Oshiro, N., Kinoshita, N., Kawano, Y., Matsuoka, Y., Bennett, V., Matsuura, Y., and Kaibuchi, K. (1999). Phosphorylation of adducin by Rho-kinase plays a crucial role in cell motility. *J Cell Biol* 145**,** 347-361.

Furukawa, N., Ongusaha, P., Jahng, W.J., Araki, K., Choi, C.S., Kim, H.J., Lee, Y.H., Kaibuchi, K., Kahn, B.B., Masuzaki, H., Kim, J.K., Lee, S.W., and Kim, Y.B. (2005). Role of Rho-kinase in regulation of insulin action and glucose homeostasis. *Cell Metab* 2**,** 119-129.

Goto, H., Kosako, H., Tanabe, K., Yanagida, M., Sakurai, M., Amano, M., Kaibuchi, K., and Inagaki, M. (1998). Phosphorylation of vimentin by Rho-associated kinase at a unique amino-terminal site that is specifically phosphorylated during cytokinesis. *J Biol Chem* 273**,** 11728-11736.

Hahmann, C., and Schroeter, T. (2010). Rho-kinase inhibitors as therapeutics: from pan inhibition to isoform selectivity. *Cell Mol Life Sci* 67**,** 171-177.

Hiroi, Y., Noma, K., Kim, H.H., Sladojevic, N., Tabit, C.E., Li, Y., Soydan, G., Salomone, S., Moskowitz, M.A., and Liao, J.K. (2018). Neuroprotection Mediated by Upregulation of Endothelial Nitric Oxide Synthase in Rho-Associated, Coiled-Coil-Containing Kinase 2 Deficient Mice. *Circ J* 82**,** 1195-1204.

Inada, H., Goto, H., Tanabe, K., Nishi, Y., Kaibuchi, K., and Inagaki, M. (1998). Rho-associated kinase phosphorylates desmin, the myogenic intermediate filament protein, at unique amino-terminal sites. *Biochem Biophys Res Commun* 253**,** 21-25.

Kaneko, T., Amano, M., Maeda, A., Goto, H., Takahashi, K., Ito, M., and Kaibuchi, K. (2000). Identification of calponin as a novel substrate of Rho-kinase. *Biochem Biophys Res Commun* 273**,** 110-116.

Kaneko, T., Maeda, A., Takefuji, M., Aoyama, H., Nakayama, M., Kawabata, S., Kawano, Y., Iwamatsu, A., Amano, M., and Kaibuchi, K. (2005). Rho mediates endocytosis of epidermal growth factor receptor through phosphorylation of endophilin A1 by Rho-kinase. *Genes Cells* 10**,** 973-987.

Kang, J.H., Asai, D., Tsuchiya, A., Mori, T., Niidome, T., and Katayama, Y. (2011). Peptide substrates for Rho-associated kinase 2 (Rho-kinase 2/ROCK2). *PLoS One* 6**,** e22699.

Kimura, K., Ito, M., Amano, M., Chihara, K., Fukata, Y., Nakafuku, M., Yamamori, B., Feng, J., Nakano, T., Okawa, K., Iwamatsu, A., and Kaibuchi, K. (1996). Regulation of myosin phosphatase by Rho and Rho-associated kinase (Rho-kinase). *Science* 273**,** 245-248.

Kosako, H., Amano, M., Yanagida, M., Tanabe, K., Nishi, Y., Kaibuchi, K., and Inagaki, M. (1997). Phosphorylation of glial fibrillary acidic protein at the same sites by cleavage furrow kinase and Rho-associated kinase. *J Biol Chem* 272**,** 10333-10336.

Koyama, M., Ito, M., Feng, J., Seko, T., Shiraki, K., Takase, K., Hartshorne, D.J., and Nakano, T. (2000). Phosphorylation of CPI-17, an inhibitory phosphoprotein of smooth muscle myosin phosphatase, by Rho-kinase. *FEBS Lett* 475**,** 197-200.

Li, Z., Dong, X., Wang, Z., Liu, W., Deng, N., Ding, Y., Tang, L., Hla, T., Zeng, R., Li, L., and Wu, D. (2005). Regulation of PTEN by Rho small GTPases. *Nat Cell Biol* 7**,** 399-404.

Lim, M.J., Choi, K.J., Ding, Y., Kim, J.H., Kim, B.S., Kim, Y.H., Lee, J., Choe, W., Kang, I., Ha, J., Yoon, K.S., and Kim, S.S. (2007). RhoA/Rho kinase blocks muscle differentiation via serine phosphorylation of insulin receptor substrate-1 and -2. *Mol Endocrinol* 21**,** 2282-2293.

Matsui, T., Maeda, M., Doi, Y., Yonemura, S., Amano, M., Kaibuchi, K., Tsukita, S., and Tsukita, S. (1998). Rho-kinase phosphorylates COOH-terminal threonines of ezrin/radixin/moesin (ERM) proteins and regulates their head-to-tail association. *J Cell Biol* 140**,** 647-657.

Ming, X.F., Viswambharan, H., Barandier, C., Ruffieux, J., Kaibuchi, K., Rusconi, S., and Yang, Z. (2002). Rho GTPase/Rho kinase negatively regulates endothelial nitric oxide synthase phosphorylation through the inhibition of protein kinase B/Akt in human endothelial cells. *Mol Cell Biol* 22**,** 8467-8477.

Mori, K., Amano, M., Takefuji, M., Kato, K., Morita, Y., Nishioka, T., Matsuura, Y., Murohara, T., and Kaibuchi, K. (2009). Rho-kinase contributes to sustained RhoA activation through phosphorylation of p190A RhoGAP. *J Biol Chem* 284**,** 5067-5076.

Nagumo, H., Ikenoya, M., Sakurada, K., Furuya, K., Ikuhara, T., Hiraoka, H., and Sasaki, Y. (2001). Rho-associated kinase phosphorylates MARCKS in human neuronal cells. *Biochem Biophys Res Commun* 280**,** 605-609.

Nakayama, M., Goto, T.M., Sugimoto, M., Nishimura, T., Shinagawa, T., Ohno, S., Amano, M., and Kaibuchi, K. (2008). Rho-kinase phosphorylates PAR-3 and disrupts PAR complex formation. *Dev Cell* 14**,** 205-215.

Nunes, K.P., Rigsby, C.S., and Webb, R.C. (2010). RhoA/Rho-kinase and vascular diseases: what is the link? *Cell Mol Life Sci* 67**,** 3823-3836.

Ohashi, K., Nagata, K., Maekawa, M., Ishizaki, T., Narumiya, S., and Mizuno, K. (2000). Rho-associated kinase ROCK activates LIM-kinase 1 by phosphorylation at threonine 508 within the activation loop. *J Biol Chem* 275**,** 3577-3582.

Ohta, Y., Hartwig, J.H., and Stossel, T.P. (2006). FilGAP, a Rho- and ROCK-regulated GAP for Rac binds filamin A to control actin remodelling. *Nat Cell Biol* 8**,** 803-814.

Ongusaha, P.P., Qi, H.H., Raj, L., Kim, Y.B., Aaronson, S.A., Davis, R.J., Shi, Y., Liao, J.K., and Lee, S.W. (2008). Identification of ROCK1 as an upstream activator of the JIP-3 to JNK signaling axis in response to UVB damage. *Sci Signal* 1**,** ra14.

Riento, K., Totty, N., Villalonga, P., Garg, R., Guasch, R., and Ridley, A.J. (2005). RhoE function is regulated by ROCK I-mediated phosphorylation. *EMBO J* 24**,** 1170-1180.

Sumi, T., Matsumoto, K., and Nakamura, T. (2001). Specific activation of LIM kinase 2 via phosphorylation of threonine 505 by ROCK, a Rho-dependent protein kinase. *J Biol Chem* 276**,** 670-676.

Surma, M., Wei, L., and Shi, J. (2011). Rho kinase as a therapeutic target in cardiovascular disease. *Future Cardiol* 7**,** 657-671.

Takefuji, M., Mori, K., Morita, Y., Arimura, N., Nishimura, T., Nakayama, M., Hoshino, M., Iwamatsu, A., Murohara, T., Kaibuchi, K., and Amano, M. (2007). Rho-kinase modulates the function of STEF, a Rac GEF, through its phosphorylation. *Biochem Biophys Res Commun* 355**,** 788-794.

Tanaka, T., Nishimura, D., Wu, R.C., Amano, M., Iso, T., Kedes, L., Nishida, H., Kaibuchi, K., and Hamamori, Y. (2006). Nuclear Rho kinase, ROCK2, targets p300 acetyltransferase. *J Biol Chem* 281**,** 15320-15329.

Vahebi, S., Kobayashi, T., Warren, C.M., De Tombe, P.P., and Solaro, R.J. (2005). Functional effects of rho-kinase-dependent phosphorylation of specific sites on cardiac troponin. *Circ Res* 96**,** 740-747.

Wallert, M.A., Hammes, D., Nguyen, T., Kiefer, L., Berthelsen, N., Kern, A., Anderson-Tiege, K., Shabb, J.B., Muhonen, W.W., Grove, B.D., and Provost, J.J. (2015). RhoA Kinase (Rock) and p90 Ribosomal S6 Kinase (p90Rsk) phosphorylation of the sodium hydrogen exchanger (NHE1) is required for lysophosphatidic acid-induced transport, cytoskeletal organization and migration. *Cell Signal* 27**,** 498-509.
